# Comparing shade tolerance measures of woody forest species

**DOI:** 10.7717/peerj.5736

**Published:** 2018-10-09

**Authors:** Jiayi Feng, Kangning Zhao, Dong He, Suqin Fang, TienMing Lee, Chengjin Chu, Fangliang He

**Affiliations:** 1 Department of Ecology, State Key Laboratory of Biocontrol, School of Life Sciences, Sun Yat-sen University, Guangzhou, China; 2 Tiantong National Station for Forest Ecosystem Research, School of Ecology and Environmental Science, East China Normal University, Shanghai, China; 3 Department of Renewable Resources, University of Alberta, Edmonton, AB, Canada

**Keywords:** Shade tolerance, Low-light abundance, Light requirement, Succession, Woody forest species

## Abstract

Shade tolerance, the minimum light requirement for plant survival, is a key trait for understanding community assembly and forest dynamics. However, it is poorly defined for tree species to date. Current methods of measuring shade tolerance vary considerably in their performance. For instance, some measures of shade tolerance are unreliable except under some specific conditions. Therefore, it is necessary to compare the performance of these methods to provide guidance of choosing appropriate shade tolerance measures in future studies. We collected a large dataset of light traits and other life history traits for 137 understory wood species in a subtropical forest and tested the performance of five commonly used shade-tolerance indices. Results showed that all the shade-tolerance measures, except the low-light abundance index, performed poorly in distinguishing and ranking shade tolerance of the tested species. The shade tolerance quantified by the low-light abundance was consistent with empirical classification of shade-tolerance/intolerance groups and successional seral stages of species. Comparison of the shade tolerance between trees of different diameter at breast height (DBH) or height classes further confirmed the reliability of low-light abundance. We conclude that low-light abundance is the most objective and practical of the five most commonly-used methods for measuring and ranking shade tolerance of understory wood species in our study forest, and likely in other forests as well. The simplicity of the method should greatly facilitate the assessment of light niche differentiation between species and thus contribute to understanding coexistence of tree species in forests.

## Introduction

Light is a fundamental resource limiting the growth and survival of plants in nature (Chazdon & Fetcher, 1984; Leuchner et al., 2012). Shade tolerance, the minimal light requirement for plant survival, is an important indicator of plant performance under different light conditions and is a key trait for understanding community assembly and forest dynamics (Bazzaz, 1979; Zavala et al., 2007; Comita & Hubbell, 2009). However, there is little consensus on how the degree of shade tolerance of woody species is quantified and hence the classification of tree species into the shade tolerant or intolerant categories (Valladares & Niinemets, 2008; Lusk & Jorgensen, 2013).

While many methods have been proposed to measure species’ degree of shade tolerance (Table 1), the evaluation of various indices has been elusive. In early studies, shade tolerance of woody plants was classified by subjectively summarizing opinions about shade tolerance of species from experienced foresters (Baker, 1949; Ellenberg, 1974). This practice relied on the qualitative observations of researchers and thus was inconsistent and difficult to categorize plants in unique categories. Moreover, qualitative observations coarsely classified species into discrete groups and thus were not able to distinguish subtle light segregation between many species (Humbert et al., 2007). Objective shade tolerance measures were later developed to incorporate other factors including plant performance or light conditions (Table 1). A simple method is to measure species’ shade tolerance from abundance distribution along a light gradient (Lorimer, 1983; Poorter & Arets, 2003). One of these abundance-based indices is to compare shade tolerance by sapling ratios in the shady environment of the target species (Poorter & Arets, 2003). The sapling ratio is defined as the ratio of the number of saplings growing in low-light environment over the total abundance of the species. While easy to implement, this method is inaccurate if the relative abundances of two species are very different (Poorter & Arets, 2003). Another abundance-based index is to use the number of stems in the shady environment (i.e., low-light abundance) of the target species to infer shade tolerance (Lorimer, 1983). To compare these indices, experiments may need to control the effect of key resources on species abundance (Craine et al., 2012) because other resource gradients may confound the comparison as light resource often varies and interacts with other environmental factors (Niinemets & Valladares, 2006).

**Table 1:** Summary on required data, advantages, disadvantages and references of methods used to measure shade tolerance of forest tree species.

Methods	Data required	Advantages	Disadvantages	Reference
Empirical classification	Subjective opinions of researchers	No field work required	Lack of standardized procedures difficult to separate shade tolerance if there are many species	Baker (1949), Ellenberg (1974)
Abundance of species along light gradient	Low-light abundance or sapling ratio	Abundance data are widely available and easy to collect	Abundance is often affected and confounded by other resources, such as drought and waterlogging	Lorimer (1983), Poorter & Arets (2003)
Demographic performance	Mortality or/and growth rates	Demographic rates are considered to be good indicators of plant’s performance in response to environment	Require temporal, sometimes long-term data for calculating demographic rates. Relationships between shade tolerance and growth/mortality rates are often not as strong	Kobe et al. (1995), Weber et al. (2017), Walters & Reich (1996), Sendall, Lusk & Reich (2016)
Light environment	Light level around target trees	Reflect the preference of actual light environment of species. Data are relatively easy to collect	Surrounding light level is often insufficient to determine light preference of species. Hard to distinguish shade tolerance if there are many species	Lusk & Reich (2000), Figueroa & Lusk (2001), Lusk et al. (2008)
Plant traits	Organ- or sub-organ-level plant traits	Functional trait database is often available	Traits often have poor predictive power for responses to environmental conditions	Valladares & Niinemets (2008), Craine et al. (2012)
Light-response curves	Light-response curves across light gradient	Describe whole plant’s performance across light gradient; accurately reflect plant’s minimum light requirement	Costly in labor	Poorter et al. (2010)
Successional seral stage	Successional scores of species	No field work required	Successional data are often not available or difficult to determine	Poorter & Arets (2003), Niinemets & Valladares (2006)

An alternative measure of shade tolerance is to consider demography (Table 1). Species demographics, especially growth and mortality, is commonly used to infer species shade tolerance (Valladares & Niinemets, 2008; Wright et al., 2010). For example, the juvenile mortality rate is used to quantify shade tolerance (Kobe et al., 1995; Weber et al., 2017). However, measuring mortality rates of juveniles in the field requires a sufficiently long-time interval (Lusk & Jorgensen, 2013) and it is sometimes difficult to identify species of dead individuals. In addition to mortality rate, the relative growth rate (RGR) is also used to measure shade tolerance. The RGR of shade tolerant species in low-light is assumed to be larger than that of intolerant species owing to their tolerance in light-limited environments (Walters & Reich, 1996; Sendall, Lusk & Reich, 2016). In contrast, experimental evidence indicated that shade intolerant species maintained a higher RGR than tolerant species irrespective of the light environment (Kitajima, 1994; Poorter, 1999), but see Baltzer & Thomas (2007a). Although there is a general interspecific tradeoff between high-light growth and low-light survival (Pacala et al., 1996; Wright et al., 2010), this tradeoff is proved to be strongly influenced by tree size (Kunstler, Coomes & Canham, 2009). Therefore, it is sometimes considered unreliable to measure shade tolerance of woody species according to relationship between high-light growth and low-light survival. In addition, the tradeoff does not seem strong enough to explain light partitioning patterns of species (Gravel et al., 2010).

Light environment (e.g., canopy openness) around target trees is often used to measure their shade tolerance (Lusk & Reich, 2000; Figueroa & Lusk, 2001; Lusk et al., 2008). Although advanced technologies (e.g., hemispherical photography and LAI-2000 Canopy Analyzer) were widely used to measure understory light environment (Jennings, Brown & Sheil, 1999; Fiala, Garman & Gray, 2006; Peng, Zhao & Xu, 2014; Zhao & He, 2016), distinguishing shade tolerance abilities between species with similar light requirement is undeveloped because light intensity of most forest understory is generally low and/or has a narrow range (Chazdon & Fetcher, 1984; Canham et al., 1990).

In addition to data on the whole plant-level performance, organ- or sub-organ-level functional traits that determine how plants interact with light are also used to infer shade tolerance of species (Valladares & Niinemets, 2008). For example, the leaf light compensation point and the leaf dark respiration rate are shown to be lower in shade tolerant species than intolerant ones (Baltzer & Thomas, 2007a; Valladares & Niinemets, 2008) and hence are supposed to be good estimators of shade tolerance of tree species. However, for several reasons organ-level and ecophysiological traits have limited capacity in classifying species’ ecological performance (Craine et al., 2012). First, the connection between traits and particular ecological performance of species may not be as close as expected (Craine et al., 2012). Second, the phenotypes are influenced by many factors and these effects could be very complicated (Houle, Govindaraju & Omholt, 2010; He et al., 2018). For example, the high plasticity of some plant traits could lead to inconsistent relationships between traits and species’ ecological niche or potential performance (Valladares et al., 2000; Sterck et al., 2013). As such, it is argued that poor results could arise if species tolerance is only estimated by organ-level or sub-organ-level traits (Wright et al., 2010; Craine et al., 2012).

Physiologically, light response curves of species can be used to deduce the minimum light requirement of species (Poorter et al., 2010). However, in order to acquire such light response curves, plants need to be exposed to various light conditions to determine the light level at which the growth of the species becomes zero. The amount of work required to determine light response curves to distinguish the shade tolerance for a large number of tree species thus makes the method impracticable. If data on the time of species colonization in succession are available, one may use it as a successional score to measure shade tolerance by assuming that earlier successional species are more shade intolerant than later successional species (Poorter & Arets, 2003; Niinemets & Valladares, 2006). However, because the observation time in most studies is not sufficiently long, successional data are often not available. Indices that incorporate multi-factors are also used to quantify shade tolerance of woody species (Poorter & Arets, 2003; Baltzer & Thomas, 2007a). The whole-plant light compensation point (WPLCP), based on understory light environments and RGR of plants, is a commonly used measure of shade tolerance in the field (Baltzer & Thomas, 2007a, 2007b; Lusk & Jorgensen, 2013). Species with the lower WPLCP are less likely to die in low light environment and are supposed to be more shade tolerant (Baltzer & Thomas, 2007a, 2007b). However, this approach requires monitoring a large number of individuals and thus is not feasible when we need to compare shade tolerance among a large number of species.

Despite multiple methods can potentially assess woody plant shade tolerance, there is a lack of consensus in the performance or adequacy of these methodologies. In this study, we compared and tested the following five measures that are commonly used to quantify shade tolerance (also see Table 1), including low-light abundance (Lorimer, 1983), sapling ratio (Poorter & Arets, 2003), mortality (Kobe et al., 1995), light environment (Lusk et al., 2008) and leaf light compensation point (LCP) measurement (Valladares & Niinemets, 2008). Given that no pre-existing objectively defined shade tolerance for species in our study site, we used the following three criteria to assess the above indices. First, the indices are consistent with an empirically documented classification of shade-tolerance/intolerance groups. Second, the indices are correlated with successional seral stages of the species. Lastly, the indices are correlated with two shade-tolerance related traits (leaf respiration *R_d_* and wood density). A good shade-tolerance index is expected to have strong correlation with these three criteria.

In addition, we tested the consistency among the different shade-tolerance measures by assessing their correlations. We also evaluated the indices by asking whether they are data parsimonious and how easy they are to use in the field. For application purposes, it is important to develop methods that are not only accurate and robust but also practically feasible.

## Materials and Methods

### Study site

The study site is located in the Heishiding Nature Reserve, a subtropical forest in Guangdong province, China (23°25′–23°27′N, 111°48′–111°55′E, elevation 150–700 m). The study area features a subtropical moist monsoon climate, with distinct wet and dry seasons. Mean annual precipitation is 1743.8 mm and mean annual relative humidity is over 80%. Mean annual temperature is 19.6 °C, with the lowest mean monthly temperature in January (about 10.6 °C) and the highest in July (28.4 °C). In 2011–2012, a 50 ha (1,000 × 500 m) stem-mapping plot was established. The plot has 237 tree and liana species. Our study site is located in the northwest part of the plot. It is a 5.2 ha (200 × 260 m) subplot and has 179 species, belonging to 115 genera and 57 families. Of these, data on 137 woody trees and shrub species (belonging to 47 families and 90 genera) were collected to test the five shade-tolerance measures in this study. Field experiment was permitted by Sun Yat–sen University.

### Measuring light environment

To measure light environment in our 5.2 ha study plot, we used an instantaneous measure of percent photosynthetic photon flux density (%PPFD) taken under overcast sky conditions to estimate the mean daily %PPFD at any microsites (after Parent & Messier, 1996). In this method, an instantaneous PPFD was defined as an instantaneous measure of PPFD made at any microsites (in the understory or above the canopy) by using a quantum sensor. The instantaneous %PPFD was calculated by dividing the understory instantaneous PPFD by an instantaneous PPFD measured at the same time above the canopy (Parent & Messier, 1996). Strong linear relationships were found between the instantaneous measure of %PPFD taken under overcast conditions and the mean daily %PPFD (Parent & Messier, 1996). Therefore, one single instantaneous measure of %PPFD taken under overcast conditions is considered to be sufficient to estimate the mean daily %PPFD for that microsite under both overcast and cloudless days (Messier & Puttonen, 1995). Thus, the instantaneous %PPFD can offer a rapid estimation of light availability for any location under the forest canopy. There were 14,365 stems of the 137 woody species with height ranging from one to five m in the understory of the 5.2 ha plot. We randomly sampled individuals (or saplings) from these stems to measure light environments above them whenever feasible. In total, light environment was measured above 8,717 stems randomly sampled. Instantaneous PPFD above each sampled sapling was measured by calibrating LI-190 quantum sensor (LI-COR, Lincoln, NE, USA). Light environment of the individual sapling was defined as the ratio of instantaneous PPFD above the stem to PPFD outside the forest plot at the same time. PPFD outside the forest was measured by a LI-190 quantum sensor installed on the top of a 70 m tall meteorological tower two km away from the 50 ha plot. All light measurements were conducted under overcast sky condition, close to sunset from July to December in 2014.

Most of the points being measured were in the closed understory (Fig. S1A). The observations showed that mean light environments of other height classes (1–4 m: 0.0209 ± 0.0222; 1–3 m: 0.0206 ± 0.0230; and 1–2 m: 0.0206 ± 0.0263) were similar with the one of 1–5 m (0.0210 ± 0.0221). Therefore, we took light measurement with trees up to five m as low-light environment in the 5.2 ha plot. In addition, the degree of light variation of all height classes was similar as well (see Fig. S1). As such, it is reasonable to assume that trees with height equal or less than five m are in the low light environment in this study. We also tested if the results from various shade-tolerance measures were consistent among the different height classes. The results confirmed the consistent assumption and supported the abundance of saplings with height ≤5 m as a reliable measure of low light condition (Table S1). To further exclude possible extreme data points, we eventually used the 10th percentile of the distribution of light environments occupied by saplings as the light environment of a species (Lusk et al., 2008).

### Quantifying low-light abundance and sapling ratios

As we defined the low-light environment as the light condition under tree height ≤5 m, the low-light abundance is the abundance of each of the 137 woody species with height ≤5 m (Lorimer, 1983). The sapling ratio is defined as the ratio of the low-light abundance over the total abundance of each species studied (Poorter & Arets, 2003). It is noteworthy that the measure of low-light abundance was robust to other height classes as well (Table S1).

### Mortality survey

Sapling mortality of each of the 137 woody species in the low-light environment was recorded according to two censuses data (the first census of the 50 ha plot was done in August 2012 and the second census was completed in December 2014). In the first census, only living stems were recorded. All saplings with which light environment had been measured were re-surveyed in December 2014 and the living status of each sapling was recorded. Saplings missing after a thorough search were recorded as death. Annual mortality estimates were then calculated for each species according to Sheil, Burslem & Alder (1995).

### Measuring functional traits

Leaf respiration (*R_d_*) and wood density are often used as reliable surrogates measuring tree species’ shade tolerance (Craine & Reich, 2005; Baltzer & Thomas, 2007a; Janse-ten Klooster, Thomas & Sterck, 2007; Nock et al., 2009). In the present work, these two functional traits were used to compare the performance of the five shade-tolerance measures that are assessed. In addition, LCP considered as one of the shade tolerance metrics in this study, and *R_d_* were measured for each of the 137 woody species with the height ranging from one to five m. Samples were located in understory characterized by low light (see Fig. S2). For species with understory abundance ≥6, six sapling individuals of each species were randomly selected. From each sampled individual, one healthy and fully developed new leaf at the top of the sapling was chosen for measuring the light-response curve. For species with understory abundance <6 individuals, all individuals were sampled. In total, 704 individuals were measured in the 5.2 ha plot. Species-level mean values of LCP were subsequently used as a shade-tolerance measure.

We compared the light environment of the measured species (%PPFD of 110 species with more than three sampled saplings) and found that only *Evodia lepta* and 45 other species showed significant differences in light environment among the total 5,995 species pairs (∼0.75%, Table S2). It indicated that most of the light environment where measured saplings were growing were comparable and photosynthetic traits (LCP and *R_d_*) measured in this condition should not have caused crucial bias in our study.

In the growing season (May–September) of 2013 and 2014, light-response curves were measured for each target leaf by using a portable photosynthetic system (LI-6400; LI-COR, Lincoln, NE, USA). The CO_2_ concentration of sample room was set to 400 μmol/(m^2^ × s) by the CO_2_ offering module (6400-01 CO_2_ Mixer). Leaf temperature was set to 25 °C and relative humidity was set to 75–85%. The gradient of PPFD was set to 2,000, 1,500, 1,000, 500, 200, 150, 100, 50, 20, 0 μmol/(m^2^ × s) with the red-blue light resource module (6400-02B LED Light Source). Measurement was processed under the automatic light-curve program. At each PPFD level 2–3min were spent to allow the leaf to reach the photosynthesis stable stage from a high light level to a low light level. So it took 30 min to measure a light-response curve for each leaf. Each target leaf was induced by a luminescence LED lamp for at least 30 min just before the operation of the automatic program. The intensity of the induced light was about 2,000 μmol/(m^2^ × s). Mitscherlich model (after Potvin, Lechowicz & Tardif, 1990) was used to fit light-response curves with the measured plant photosynthetic data:

$$A = {A_{{\rm{max}}}} \times \left[ {1-{e^{-{\rm \phi} \times \left({{\rm{PPFD-LCP}}} \right)}}} \right],$$

where *A*_max_ is the maximum rate of photosynthesis and ϕ represents the apparent quantum yield. LCP corresponds to the photosynthetic light compensation point, PPFD is the photosynthetic photo flux density a leaf receives, and *A* refers to the net photosynthesis at any light level (Potvin, Lechowicz & Tardif, 1990). Net photosynthesis and PPFD data were used to fit the Mitscherlich equation and the model was parameterized by using the function “nls” in the R software (R Development Core Team, 2017). Respiratory rate (*R_d_*) is defined as the photosynthesis rate when no light resource is available to photosynthesis. We calculated *R_d_* from the Mitscherlich equation by setting the PPFD value to 0 based on the values of other parameters that have been evaluated from the Mitscherlich equation (Potvin, Lechowicz & Tardif, 1990).

Wood density was measured for 184 species in the 50 ha plot during June and August in 2014. Of these 184 species, 132 were found in the 5.2 ha subplot and were included in the present study. For species with abundance of more than 20 individuals, 20 individuals of each of such species were randomly selected. For rare species (with abundance ≤20), wood density for every individual tree were measured. For each selected tree, outer crown twigs of non-current-year were harvested to measure wood density. For trees with DBH ≥6 cm, in addition to the crown twig samples, a three to five cm long trunkwood core was also extracted by using a borer with the four to five mm caliber (He & Deane, 2016). The mean value of twig and trunk wood density across individuals represented the species wood density.

### Empirical data on functional groups

We compiled data on successional seral stages and shade-tolerance groups of the species in question. Species successional seral stages and shade-tolerance/intolerance groups were summarized with the reference to *Flora of China* (http://www.efloras.org/) and Zhou et al. (1999) (Table S3). Zhou et al. (1999) focused on the successional seral stages of the species of the Heishiding Nature Reserve, in which species that reached maximum abundance by 35 years after clear-cut were considered as early successional species, and species reaching maximum abundance between 35 and 60 years after clear-cut were considered as middle successional species, and species reaching maximum abundance after 100 years of clear-cut were later stages species. Furthermore, for species that were not included in Zhou et al. (1999) but were described as “pioneer species” in *Flora of China*, they were classified as early successional species. In total, successional seral stages for 59 species were classified (Table S3). In addition to successional seral stages, we also compiled data on species shade-tolerance and shade-intolerance groups according to the description in *Flora of China* and Zhou et al. (1999). Species described as “heliophyte,” “living in high light environment,” or “shade intolerant” were assigned to the group of shade-intolerance, while species described as “mesophyte,” “living in shady environment” or “shade tolerant” were assigned to the group of shade-tolerance. Species with controversial or ambiguous descriptions about shade-tolerance ability were excluded to minimize misclassification. In total, we were able to classify 22 species into either shade-tolerance or intolerance group (Table S3). The classification of shade-tolerance and intolerance groups more accurately describes species’ shade tolerance than successional seral stages. The successional seral stage is related to shade tolerance, but the relationship is less certain. Although it is a general trend that earlier successional species are also less shade tolerant, light demanding species could also be non-pioneer species which reach maximal abundance in the middle and later successional stages (Poorter & Arets, 2003). The compiled data of shade-tolerance groups and successional seral stages were used to test whether the first two proposed criteria assessing shade-tolerance indices were met, respectively.

### Robustness test of the best shade-tolerance measure

We tested the robustness of the “best” shade tolerance measure (low-light abundance) by defining it using different DBH and different height classes. To do that, the low-light abundance measure was recalculated using four DBH classes: 1–2 cm, 1–3 cm, 1–4 cm and 1–5 cm in diameter. Within each DBH class cutoff, low-light abundance was still defined as the abundance of target species with height ≤5 m. Similarly, we recalculated the index at different height classes: 1–2 m, 1–3 m, 1–4 m and 1–5 m tall. Within each height class cutoff, low-light abundance was defined as the abundance of target species with height ≤2 m (for 1–2 m class cutoff), 3 m (for 1–3 m class cutoff), 4 m (for 1–4 m class cutoff) and 5 m (for 1–5 m class cutoff), respectively. Results of different height classes can also help support our assumption of using height ≤5 m as the low light condition in our study.

### Statistical analysis

In this study, the Spearman’s rank correlation test was used to assess the association between functional groups (or functional traits) and the shade-tolerance indices including low-light abundance, sapling ratio, mortality rate, light environment and LCP. The Wilcoxon rank test and Kruskal–Wallis test were used to test if shade tolerance measured by shade-tolerance indices between different functional groups is different. The correlation of species’ shade tolerance measured by different indices was assessed by the Spearman’s rank correlation. The relationships of shade tolerance measured by low-light abundance between different DBH or height classes were assessed by the Pearson correlation test. All analyses were implemented using the R software (R Development Core Team, 2017).

## Results

### Performance of different shade-tolerance measures

The results in Table 2 showed that the low-light abundance was the only measure that forms significant correlation with successional seral stages and the two functional traits of species. There was a significant difference in low-light abundance between early and later successional stage and between shade intolerant and tolerant groups (Table 2). The sapling ratio showed no relationship with successional stages of species (Kruskal test, *P* > 0.05; Table 2) but displayed a significant difference between shade intolerant and shade tolerant groups (Wilcoxon rank sum test, *P* < 0.05). Mortality and LCP of species were not distinguishable between shade intolerant and tolerant species and between different successional stages. Mortality only showed a signal in relationship with wood density. LCP only showed a strong correlation with *R_d_* (Table 2). Light environment showed a significant correlation with successional seral stages and wood density (Table 2) but showed no difference between different successional stages (Kruskal test, *P* > 0.05; Table 2) nor between shade intolerant and shade tolerant groups (Wilcoxon rank sum test, *P* > 0.05; Table 2).

**Table 2:** Relationships between shade tolerance measures and functional groups (or functional traits), and the difference in measures between shade-tolerance/intolerance groups (or different successional stages).

Shade-tolerance measures	Association with functional groups or functional traits				Difference in the value of a measure between shade-tolerance/intolerance groups and between different successional seral stages	
	Successional seral stages	Shade-tolerance/intolerance groups	*R_d_*	Wood density	Successional seral stages	Shade-tolerance/intolerance groups
	*n* = 59	*n* = 22	*n* = 137	*n* = 132	*n* = 59	*n* = 22
Low-light abundance	0.51***	0.85***	−0.11**	0.28***	Early < later***	Intolerant < tolerant***
Sapling ratio	ns	ns	−0.09*	−0.10***	ns	Intolerant < tolerant*
Mortality	ns	ns	ns	0.05**	ns	ns
Light environment	−0.27*	ns	ns	−0.12***	ns	ns
LCP	ns	ns	0.46***	ns	ns	ns

**Notes:**

The Spearman’s rank correlation was used to assess the association between functional groups (or functional traits). Difference in measures between shade-tolerance/intolerance groups (or different successional stages) was tested by the Wilcoxon rank test (Kruskal–Wallis test). Data on successional seral stages (59 species) and shade-tolerance/intolerance groups (22 species) are presented in the Appendix Table S2. *R_d_* is mean species value of leaf respiration. Wood density is mean species value. *n* is the number of species.

*** *P* ≤ 0.001;

** *P* ≤ 0.01;

* *P* ≤ 0.05, and

ns is for non-significant difference.

The correlations among the five shade-tolerance measures were shown in Table 3. The low-light abundance measure had strong correlations with all other measures except LCP. This result further indicates the utility of low-light abundance as a shade-tolerance measure. The light environment also showed a significant correlation with mortality, while the rest did not show any correlations with other shade-tolerance measures.

**Table 3:** Correlations among different measures of species shade tolerance.

Shade-tolerance measures	Low-light abundance	Sapling ratio	Mortality	Light environment
Sapling ratio	0.25*			
Mortality	0.45**	ns		
Light environment	−0.52**	ns	−0.37**	
LCP	ns	ns	ns	ns

**Notes:**

** *P* ≤ 0.001;

* *P* ≤ 0.01;

ns is for non-significant difference.

### Robustness of low-light abundance

**Figure 1:**
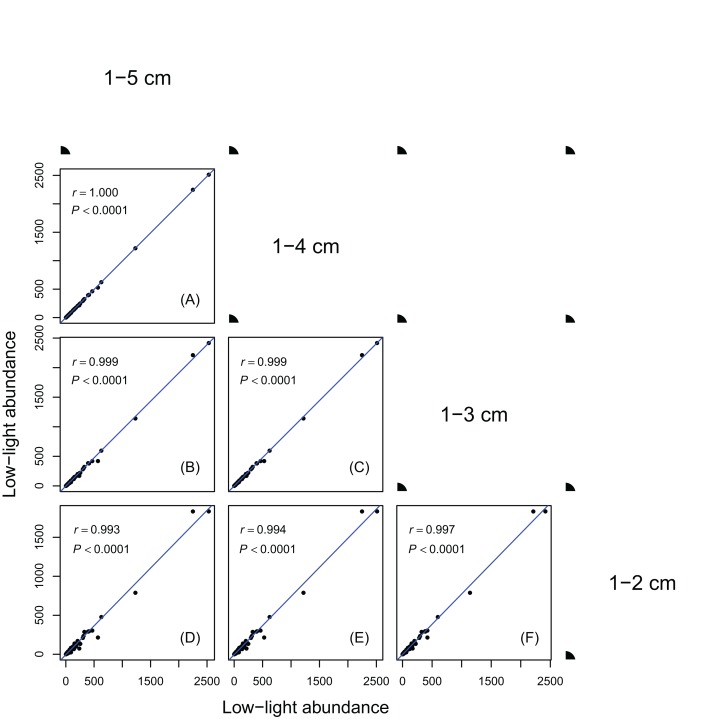
Relationships between low-light abundances counted at different DBH class cutoffs. Relationships between low-light abundances counted at 1–5 cm and 1–4 cm class cutoffs (A); 1–5 cm and 1–3 cm class cutoffs (B); 1–4 cm and 1–3 cm class cutoffs (C); 1–5 cm and 1–2 cm class cutoffs (D); 1–4 cm and 1–2 cm class cutoffs (E); 1–3 cm and 1–2 cm class cutoffs (F). There are 137 species in each DBH class cutoffs. Relationships were assessed by Pearson’s correlation coefficients. Each point represents a species value of low-light abundance counted at corresponding DBH class cutoffs. Low-light abundance is the abundance of target species with height ≤5 m in each DBH class. Species ranks of low-light abundances were highly consistent across different DBH classes.

**Figure 2:**
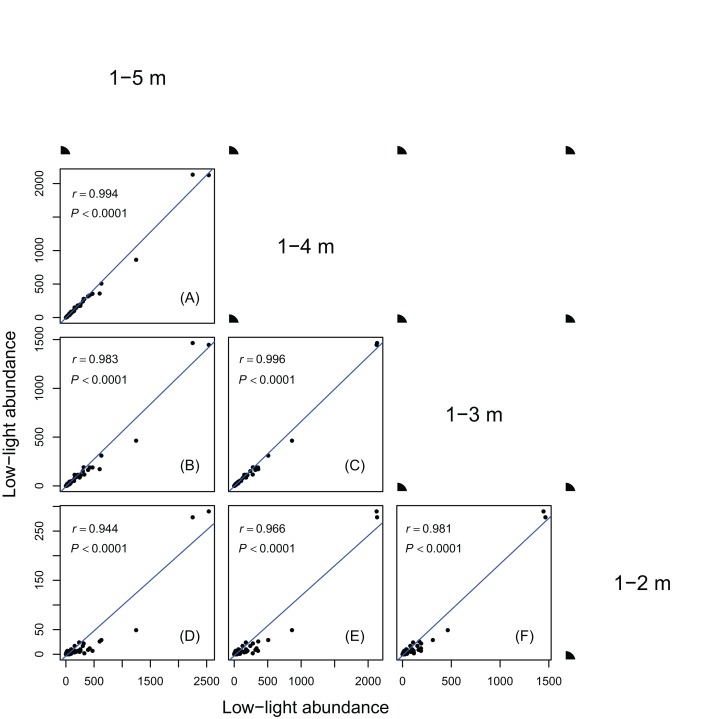
Relationships between low-light abundances counted at different height class cutoffs. Relationships between low-light abundances counted at 1–5 m and 1–4 m class cutoffs (A); 1–5 m and 1–3 m class cutoffs (B); 1–4 m and 1–3 m class cutoffs (C); 1–5 m and 1–2 m class cutoffs (D); 1–4 m and 1–2 m class cutoffs (E); 1–3 m and 1–2 m class cutoffs (F). There are 137 species in each height class cutoffs. Relationships were assessed by Pearson’s correlation coefficients. Each point represents a species value of low-light abundance counted at corresponding height class cutoffs. Low-light abundance is the abundance of target species with height ≤5 m (for 1–5 m class cutoff), 4 m (for 1–4 m class cutoff), 3 m (for 1–3 m class cutoff) and 2 m (for 1–2 m class cutoff). Species ranks of low-light abundances were highly consistent between different height classes.

Results in Figs. 1 and 2 showed species ranks of shade tolerance were highly consistent across different DBH classes and between different height classes. This means that the rank of species low-light abundance changed very little regardless of DBH classes or height classes. This ensures the robustness of the low-light abundance when used to quantify species shade tolerance.

## Discussion

To qualify as a good shade-tolerance measure, it should at least be able to correctly rank the degree of species shade tolerance, even if it could not accurately measure shade tolerance. A measure should also be data parsimonious, simple to use and easy to interpret. Our results show that the low-light abundance was the most robust shade-tolerance index. It met all three criteria proposed in the Introduction: having strong correlations with empirically documented shade tolerance data, successional seral stages and shade-tolerance related functional traits (*R_d_* and wood density) (Table 2).

The low-light abundance was useful to distinguish the species with different shade tolerance capacities, because it was consistent with the classification results of shade-tolerance/intolerance groups that were based on long-term experience of experts and experimental verification (Poorter, Bongers & Bongers, 2006; Craine et al., 2012). Due to the lack of commonly accepted data on shade tolerance, species successional seral data are often used as an important proxy to identify shade tolerance of species (Niinemets & Valladares, 2006). This is done by assuming that later successional species are more shade-tolerant than earlier successional species (Bazzaz, 1979; Denslow & Guzman, 2000). As such, we consider the correlation with successional stages to be a particularly important criterion for assessing the performance of any shade tolerance measure. By this standard, the low-light abundance was the only measure that correctly described shade tolerance of the species in our study site (Table 2). The performance of a shade-tolerance measure can also be assessed by its relationship with functional traits relevant to species’ shade tolerance. Leaf *R_d_* is low for shade tolerant species and high for intolerant species (Craine & Reich, 2005; Tsvuura et al., 2010) and it is often used as a reliable surrogate measuring tree species’ shade tolerance (Craine & Reich, 2005; Baltzer & Thomas, 2007a). Wood density is similarly used as a proxy for species shade tolerance (Janse-ten Klooster, Thomas & Sterck, 2007; Nock et al., 2009). The low-light abundance showed significant correlations with these two functional traits, supporting this measure, although the correlation with *R_d_* was relatively weak (Table 2). In addition to the significant correlations that low-light abundance had with successional seral stages and functional traits, low-light abundance also showed consistently significant correlations with most of the shade-tolerance measures (Table 3). This result further supports the reliability of the low-light abundance measure.

Light environment, mortality rate and LCP were poor shade-tolerance measures as they cannot differentiate species between shade-tolerance group and shade-intolerance group (Table 2). They were even less likely to distinguish shade tolerance for species growing in a similar low-light environment. Another evidence that mortality rate and LCP were incapable of measuring shade-tolerance in our study is that they only met one of the three criteria (i.e., criterion 3—correlated with shade-tolerance related traits; see Introduction). Light environment and sapling ratio, meeting two of our criteria, performed better than other measures but did not out-perform the low-light abundance. The sapling ratio showed no correlation with successional seral stages although it had a strong relationship with shade-tolerance group and shade-intolerance group (Table 2). Poorter & Arets (2003) suggested the sapling ratio could be only used in the situation where the abundances of two species were similar when comparing shade tolerance. This suggestion also applies to our study. For instance, *Melastoma affine* in our study has 100% sapling ratio, while sapling ratio for *Cryptocarya concinna* is 80.11% but *C. concinna* is a later successional species that is shade tolerant while *M. affine* is a shade-intolerant earlier successional species (Table S3).

Data parsimonious, simple to use and easy to interpret are also important, practical criteria for assessing the usefulness of shade-tolerance measures. Cost, logistic support, and the amount of observation time required in the field are some of the practical constraints that must be considered when determining which metric to use. In this respect, the low-light abundance and the sapling ratio emerged as good candidates as their data are widely available and easy to collect.

Although mortality data seem easy to collect, it requires a sufficiently long-time interval to collect. In our study site, no mortality was observed in more than half of the species (71 out of 137 species) during the two censuses. Therefore, it is possible that the time interval between the two censuses is not long enough, which results in no correlation between mortality and the classification of shade-tolerance/intolerance groups or successional seral stages.

Measurement of the light environment for species depends on the equipment used for measuring light and is also strongly subject to the time when the measurement is taking place. Forest irradiance varies greatly at several different time scales (within a day, day-to-day, seasonal, and year-to-year) (Canham et al., 1990; Jennings, Brown & Sheil, 1999). Spatial variation of light within a forest (sunflecks) also varies hugely (Way & Pearcy, 2012). Hence, the snapshot measure of forest light environment is likely not a reliable measure of shade tolerance of species. The lack of the correlation between light environment and species groups or functional traits in our study could be partly due to the difficulty in accurately quantifying the understory light availability.

Although functional traits can be closely related with species’ shade tolerance, most functional traits (e.g., LCP) are considered to be highly plastic (Valladares et al., 2000; Sterck et al., 2013) and hence may show different values across space and time. Therefore, trait data should always be collected from the specific community where shade tolerances of species are evaluated.

The robustness of an index is important for obtaining consistent results when applying the index in different situations. As shown in Figs. 1 and 2, the low-light abundances were very consistent across different DBH classes and between different height classes, indicating its robustness. The consistent results between different height cutoffs also showed the reliability of using height ≤5 m as a measure of low light condition.

Although the low-light abundance as a shade tolerance metric is reliable, easy to use and intuitive to interpret, the measure does come with some limitations. This method is most likely to be successful when data are available from species in a fairly homogeneous environment. Species abundance distribution along the light axis could be jointly affected by light requirement and other stresses (Craine et al., 2012). Therefore, the use of this measure requires light to act as a primary factor dominating species’ survival in a community. This problem could also handicap the use of other methods (sapling ratio, mortality and LCP included) (Valladares & Niinemets, 2008). For instance, drought and waterlogging are another two important and widespread factors affecting dynamics and distribution of tree species populations and are found inversely associated with shade tolerance (Niinemets & Valladares, 2006). These factors could also affect the tree species populations in our forest and may explain why the correlation between successional seral stages and three shade-tolerance measures was insignificant (Table 2). A future improvement on shade-tolerance measures may be to integrate the low-light abundance with related environmental factors or life history traits.

To the best of our knowledge, the present work is first at comparing methods to assess shade tolerance of woody species using a large tree dataset. The large sample size and the integrity of dataset in one community ensure the reliability of the results. For example, the large sample size allows for comparisons across size classes, otherwise it would be impossible. In addition, it is unprecedented to integrate so many species into a method comparison study to explore the best approaches to present shade tolerance for tree species.

## Conclusion

Our results indicated that low-light abundance is the most objective and practical measure in the five commonly used methods for measuring and ranking shade tolerance in our study forest. The simple-to-use of the method should be useful for assessing light niche differentiation of species and thus contributes to understanding coexistence of tree species in forests.

## Supplemental Information

10.7717/peerj.5736/supp-1 Supplemental Information 1 Fig. S1. Frequency distribution of light environment of saplings in different height class cutoffs: (A) 1–5 m class cutoff; (B) 1–4 m class cutoff; (C) 1–3 m class cutoff and (D) 1–2 m class cutoff. Mean, range, SD and *N* are the mean, range, standard deviation and the sampled number of light environment of all sampling trees in the corresponding class cutoff, respectively. Mean light environment of different height class cutoffs was similar. The degree of light variation of all height class cutoffs was similar. Light variation of 1–2 m class cutoff was higher than other classes largely due to the narrower range of light, but sample size was small in this case.

10.7717/peerj.5736/supp-2 Supplemental Information 2 Fig. S2. Frequency distribution of light environment for the individuals that were sampled for functional traits, (A) *LCP* and *R_d_*, (B) wood density. Mean, range, SD and *N* are the mean, range, standard deviation and sample number of light environment.

10.7717/peerj.5736/supp-3 Supplemental Information 3 Table S1. Relationships of shade-tolerance measures counted at different height class cutoffs. Relationships were assessed by Pearson’s correlation coefficients. Consistent results between different height class cutoffs confirmed the reasonability of assuming height ≤5 m as low light condition. Height class cutoff 1-2 m showed lower correlations with other class cutoffs, perhaps due to the small sample size. *n* is the number of species. ***P ≤ 0.001; **P ≤ 0.01; and *P ≤ 0.05.

10.7717/peerj.5736/supp-4 Supplemental Information 4 Table S2. Percent photosynthetic photon flux density (%PPFD) of each species. Data was taken on the top of saplings of the 137 woody species with height ranging 1–5 m in the understory of the 5.2 ha plot under overcast sky condition. *Sample size* is the number of saplings which light environment was measured. Species differences of %PPFD were tested by the multiple comparison with Kruskal-Wallis by using adjusted “holm” *P*-values. *P* < 0.05. Items with different superscriptletters (“a” and “b”) in column %PPFD indicate a significant difference in %PPFD between the two species. In summary, only *Evodia lepta* and 45 other species presented significant difference in growing light environment.

10.7717/peerj.5736/supp-5 Supplemental Information 5 Table S3. Species successional seral stages and shade-tolerance/intolerance groups compiled from *Flora of China* and Zhou et al. (1999).

10.7717/peerj.5736/supp-6 Supplemental Information 6 Dataset 1. Tree height and DBH of all individuals of the studied 137 species in the 5.2 ha study plot. Raw data that applied to quantify low-light abundance and sapling ratio of each species.

10.7717/peerj.5736/supp-7 Supplemental Information 7 Dataset 2. Sapling mortality of 137 species with height ranging 1–5 m in the 5.2 ha study plot. Raw data. *Mortality* is the annual mortality of each species. *n0* is stem number of each species in the first census (in August 2012). *n1* is stem number of each species in the second census (in December 2014).

10.7717/peerj.5736/supp-8 Supplemental Information 8 Dataset 3. Light intensity and status (death or alive at December 2014) of every sapling with height ranging 1-5 m of the 137 species in the 5.2 ha study plot. Raw data. *Light* is the Light environment of the individual sapling. *Forest* is light intensity on the top of the stem. *Open* is light intensity outside the forest at the same moment. *Status* is the status when the saplings were re-surveyed in December 2014: “0” for alive, “1” for death. *Tag* is the tag number of the saplings. There are 8717 samplings in total.

10.7717/peerj.5736/supp-9 Supplemental Information 9 Dataset 4. Leaf photosynthetic light compensation point (*LCP*) and respiration (*R_d_*) of the 137 species in the 5.2 ha study plot. Raw data.

10.7717/peerj.5736/supp-10 Supplemental Information 10 Dataset 5. Wood density of 132 species found in the study 5.2 ha subplot. Raw data. *Tag* is the tag number of the saplings. In total, 3523 stems were measured.

10.7717/peerj.5736/supp-11 Supplemental Information 11 Dataset 6. Results of mean test of light environment (%PPFD) of species. There were 5995 pairs of species, including 110 data enough species in the comparison (27 species did not have enough light environment data and were excluded in the comparison analysis). Species differences were test by multiple comparison with Kruskal-Wallis by using the function “kruskal” with adjust “holm” *P*-values in R project.

## Additional Information and Declarations

### Field Study Permissions

The following information was supplied relating to field study approvals (i.e., approving body and any reference numbers): Field experiments were approved by Sun Yat-sen University.

### Data Availability

The following information was supplied regarding data availability: The raw datasets are provided as Supplemental Files.
