# Supplementary figures and images for: Comparing shade tolerance measures of woody forest species
Source: PeerJ. 2018 Oct 9;6:e5736. doi: 10.7717/peerj.5736 (PMC6183557; doi:10.7717/peerj.5736)

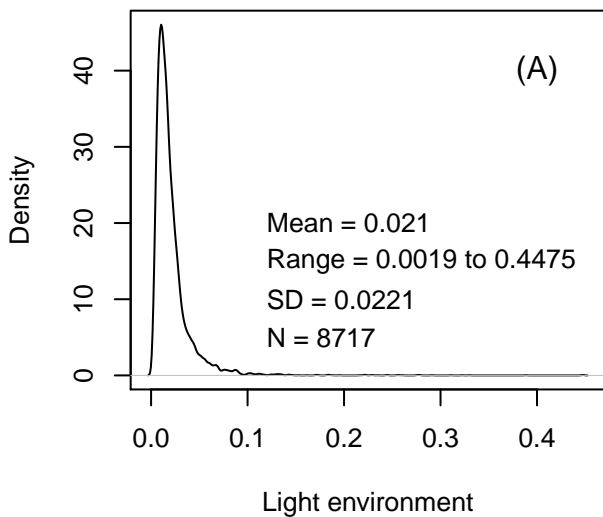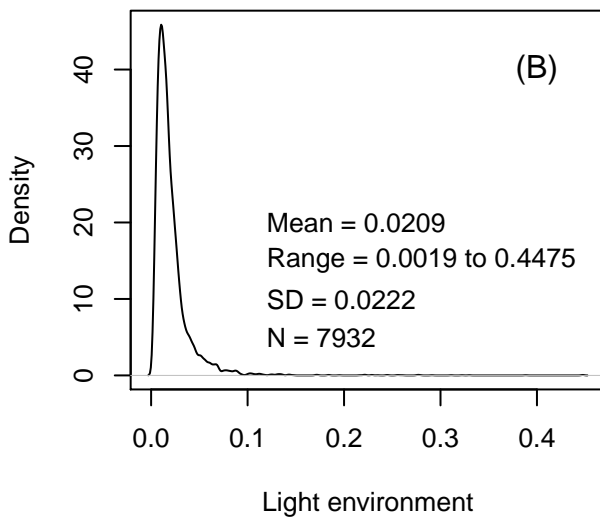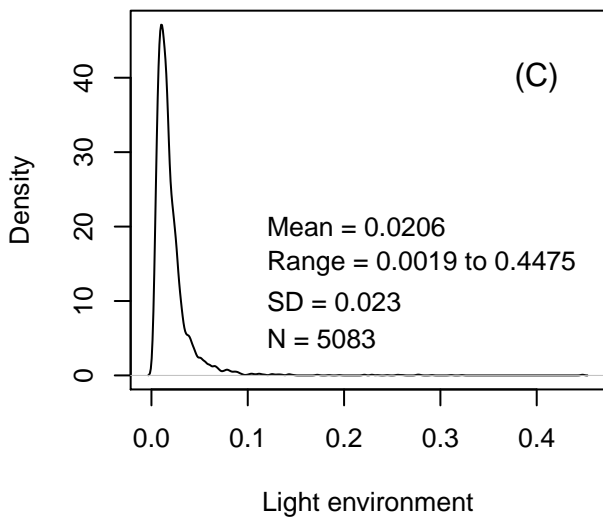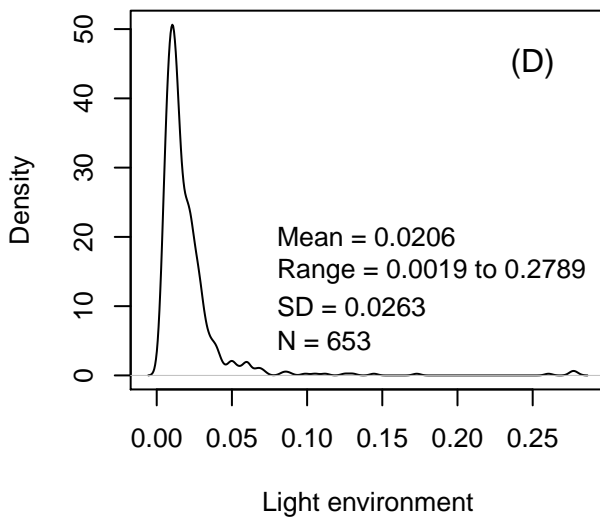

Supplement: Supplemental Information 1 — Mean, range, SD and N are the mean, range, standard deviation and the sampled number of light environment of all sampling trees in the corresponding class cutoff, respectively. Mean light environment of different height class cutoffs was similar. The degree of light variation of all height class cutoffs was similar. Light variation of 1–2 m class cutoff was higher than other classes largely due to the narrower range of light, but sample size was small in this case. [file peerj-06-5736-s001.pdf]

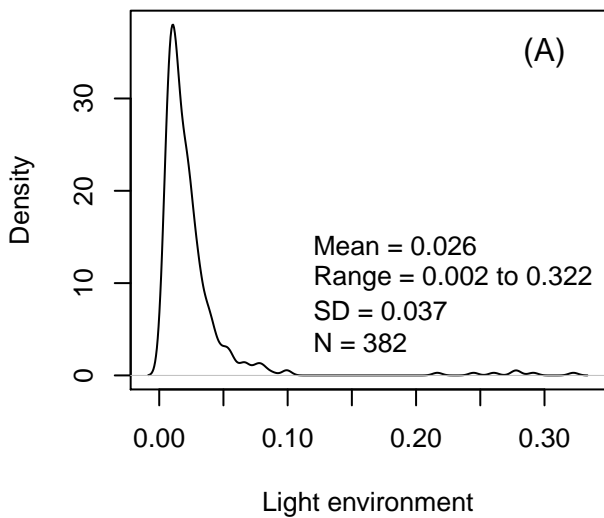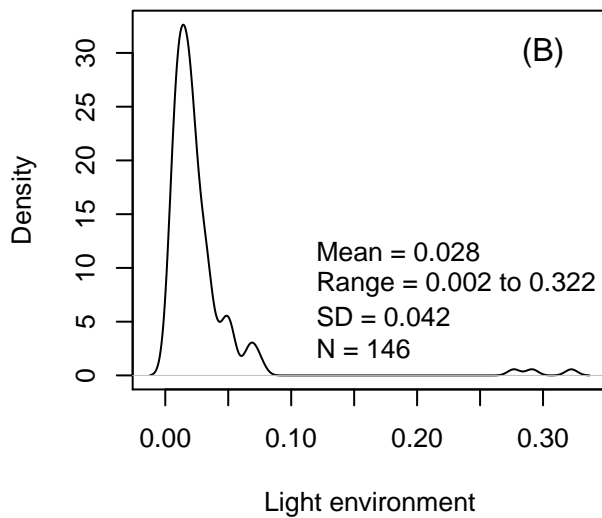

Supplement: Supplemental Information 2 — Mean, range, SD and N are the mean, range, standard deviation and sample number of light environment. [file peerj-06-5736-s002.pdf]
