# Supplementary material for: Comparing shade tolerance measures of woody forest species
Source: PeerJ. 2018 Oct 9;6:e5736. doi: 10.7717/peerj.5736 (PMC6183557; doi:10.7717/peerj.5736)
Supplement: Supplemental Information 3 — Relationships were assessed by Pearson’s correlation coefficients. Consistent results between different height class cutoffs confirmed the reasonability of assuming height ≤5 m as low light condition. Height class cutoff 1-2 m showed lower correlations with other class cutoffs, perhaps due to the small sample size. n is the number of species. ***P ≤ 0.001; **P ≤ 0.01; and *P ≤ 0.05. [file peerj-06-5736-s003.docx]

| **Shade-tolerance measure** | **Height class** | **1-5 m** | **1-4 m** | **1-3 m** |
| --- | --- | --- | --- | --- |
| Sapling ratio | 1-4 m | 0.963*** (*n*=135) |  |  |
|  | 1-3 m | 0.791*** (*n*=133) | 0.881*** (*n*=133) |  |
|  | 1-2 m | 0.274* (*n*=83) | 0.368*** (*n*=83) | 0.570*** (*n*=83) |
| Mortality | 1-4 m | 0.994*** (*n*=134) |  |  |
|  | 1-3 m | 0.830*** (*n*=129) | 0.823*** (*n*=129) |  |
|  | 1-2 m | 0.327** (*n*=68) | 0.370** (*n*=68) | 0.421*** (*n*=68) |
| Light environment | 1-4 m | 0.9997*** (*n*=134) |  |  |
|  | 1-3 m | 0.914*** (*n*=129) | 0.914*** (*n*=129) |  |
|  | 1-2 m | 0.687*** (*n*=69) | 0.687*** (*n*=69) | 0.707*** (*n*=69) |
| *LCP* | 1-4 m | 0.836*** (*n*=132) |  |  |
|  | 1-3 m | 0.780*** (*n*=121) | 0.888*** (*n*=121) |  |
|  | 1-2 m | 0.618*** (*n*=29) | 0.663*** (*n*=29) | 0.681*** (*n*=29) |
