# Supplementary material for: Comparing shade tolerance measures of woody forest species
Source: PeerJ. 2018 Oct 9;6:e5736. doi: 10.7717/peerj.5736 (PMC6183557; doi:10.7717/peerj.5736)
Supplement: Supplemental Information 4 — Data was taken on the top of saplings of the 137 woody species with height ranging 1–5 m in the understory of the 5.2 ha plot under overcast sky condition. Sample size is the number of saplings which light environment was measured. Species differences of %PPFD were tested by the multiple comparison with Kruskal-Wallis by using adjusted “holm” P-values. P < 0.05. Items with different superscriptletters (“a” and “b”) in column %PPFD indicate a significant difference in %PPFD between the two species. In summary, only Evodia lepta and 45 other species presented significant difference in growing light environment. [file peerj-06-5736-s004.docx]

| **Species** | **%PPFD (Mean ± SD)** | **Sample size** |
| --- | --- | --- |
| *Adina pilulifera* | 0.0175 ± 0.0192 | 2 |
| *Alniphyllum fortunei* | 0.3225 | 1 |
| *Altingia chinensis* | 0.0288 ± 0.0229^a^ | 21 |
| *Antidesma venosum* | 0.0213 ± 0.0164^a^ | 221 |
| *Ardisia elegans* | 0.0271 ± 0.0279^a^ | 22 |
| *Ardisia quinquegona* | 0.0201 ± 0.0156^a^ | 1694 |
| *Artocarpus hypargyreus* | 0.007 | 1 |
| *Artocarpus styracifolius* | 0.0206 ± 0.0152^a^ | 143 |
| *Beilschmiedia fordii* | 0.0185 ± 0.0105 | 21 |
| *Buxus megistophylla* | 0.0081 | 1 |
| *Callicarpa peichieniana* | 0.0206 ± 0.0169^a^ | 41 |
| *Calophyllum membranaceum* | 0.0106 | 1 |
| *Camellia caudata* | 0.0204 ± 0.0219^a^ | 248 |
| *Camellia semiserrata* | 0.0204 ± 0.0163 | 11 |
| *Camellia sinensis* | 0.0425 ± 0.0282^a^ | 6 |
| *Canarium album* | 0.0231 ± 0.0094 | 8 |
| *Carallia brachiata* | 0.0318 ± 0.0287 | 4 |
| *Casearia villilimba* | 0.0165 ± 0.0095 | 26 |
| *Castanopsis carlesii* | 0.0163 ± 0.0139 | 31 |
| *Castanopsis fabri* | 0.0312 ± 0.0206 | 3 |
| *Castanopsis fissa* | 0.0304 ± 0.0170^a^ | 10 |
| *Castanopsis fordii* | 0.0050 | 1 |
| *Castanopsis hystrix* | 0.0366 ± 0.0738 | 10 |
| *Castanopsis nigrescens* | 0.0885 | 1 |
| *Cinnamomum appelianum* | 0.0591 ± 0.0760 | 7 |
| *Cinnamomum burmanni* | 0.0184 ± 0.0118 | 7 |
| *Cinnamomum porrectum* | 0.0241 ± 0.0148 | 6 |
| *Corylopsis sinensis* | 0.0353 ± 0.0223^a^ | 11 |
| *Craibiodendron stellatum* | 0.0172 ± 0.0131 | 7 |
| *Cryptocarya concinna* | 0.0202 ± 0.0222^a^ | 1600 |
| *Cyclobalanopsis bambusaefolia* | 0.0216 ± 0.0216^a^ | 55 |
| *Cyclobalanopsis chungii* | 0.0295 ± 0.0271 | 7 |
| *Cyclobalanopsis fleuryi* | 0.0117 | 1 |
| *Cyclobalanopsis hui* | 0.0201 ± 0.0191 | 71 |
| *Daphniphyllum oldhami* | 0.0235 ± 0.0160 | 2 |
| *Dendrobenthamia hongkongensis* | 0.0142 ± 0.0116 | 3 |
| *Diospyros morrisiana* | 0.0213 ± 0.0108^a^ | 20 |
| *Diospyros strigosa* | 0.0212 ± 0.0180 | 5 |
| *Diplospora dubia* | 0.0244 ± 0.0217 | 20 |
| *Distylium racemosum* | 0.0201 ± 0.0206^a^ | 562 |
| *Elaeocarpus chinensis* | 0.0245 ± 0.0183 | 5 |
| *Elaeocarpus decipiens* | 0.0195 ± 0.0115 | 17 |
| *Engelhardtia fenzlii* | 0.0260 ± 0.0362^a^ | 51 |
| *Engelhardtia roxburghiana* | 0.0097 | 1 |
| *Enkianthus serrulatus* | 0.0149 ± 0.0042 | 3 |
| *Eriobotrya fragrans* | 0.0359 ± 0.0196^a^ | 11 |
| *Euonymus laxiflorus* | 0.0163 ± 0.0125 | 48 |
| *Eurya hebeclados* | 0.0228 ± 0.0170^a^ | 39 |
| *Eurya nitida* | 0.0103 ± 0.0005 | 2 |
| *Evodia lepta* | 0.0082 ± 0.0083^b^ | 24 |
| *Ficus erecta Thunb. Var. beecheyana* | 0.029 | 1 |
| *Ficus hirta* | 0.0203 ± 0.0114 | 7 |
| *Ficus hispida* | 0.0081 | 1 |
| *Ficus variolosa* | 0.0200 ± 0.0239 | 6 |
| *Fortunella hindsii* | 0.021 | 1 |
| *Garcinia multiflora* | 0.0249 ± 0.0231 | 16 |
| *Gardenia jasminoides* | 0.0177 ± 0.0133 | 107 |
| *Glochidion eriocarpum* | 0.0121 ± 0.0051 | 3 |
| *Glochidion wrightii* | 0.0168 ± 0.0193 | 2 |
| *Hartia villosa* | 0.0233 ± 0.0128 | 8 |
| *Heteropanax brevipedicellatus* | 0.0175 ± 0.0095 | 17 |
| *Homalium cochinchinense* | 0.0227 ± 0.0240 | 6 |
| *Ilex dasyphylla* | 0.0205 ± 0.0063 | 2 |
| *Ilex memecylifolia* | 0.0236 ± 0.0197^a^ | 32 |
| *Ilex pubilimba* | 0.0129 ± 0.0078 | 14 |
| *Ilex rotunda* | 0.0292 ± 0.0230 | 2 |
| *Ilex subficoidea* | 0.0313 ± 0.0205^a^ | 12 |
| *Ilex triflora* | 0.0192 ± 0.0120 | 7 |
| *Illicium dunnianum* | 0.0184 ± 0.0129 | 12 |
| *Itea chinensis* | 0.0252 ± 0.0225^a^ | 63 |
| *Ixonanthes chinensis* | 0.0357 ± 0.0470 | 10 |
| *Lasianthus chinensis* | 0.0172 ± 0.0031 | 3 |
| *Laurocerasus phaeosticta* | 0.0251 ± 0.0373^a^ | 73 |
| *Lindera chunii* | 0.0205 ± 0.0161^a^ | 79 |
| *Lindera metcalfiana* | 0.0228 | 1 |
| *Lithocarpus calophyllus* | 0.0215 ± 0.0145^a^ | 40 |
| *Lithocarpus litseifolius* | 0.0189 ± 0.0168 | 8 |
| *Lithocarpus lohangwu* | 0.0191 ± 0.0153 | 21 |
| *Lithocarpus uvariifolius* | 0.0227 ± 0.0220^a^ | 91 |
| *Litsea acutivena* | 0.0196 ± 0.0153^a^ | 123 |
| *Litsea elongata* | 0.0172 ± 0.0110^a^ | 211 |
| *Litsea greenmaniana* | 0.0175 ± 0.0153 | 30 |
| *Litsea rotundifolia Hemsl. var. oblongifolia* | 0.0187 ± 0.0074^a^ | 24 |
| *Machilus breviflora* | 0.0233 ± 0.0189^a^ | 170 |
| *Machilus chinensis* | 0.0179 ± 0.0038 | 3 |
| *Machilus litseifolia* | 0.0201 | 1 |
| *Machilus velutina* | 0.0256 ± 0.0275^a^ | 51 |
| *Madhuca hainanensis* | 0.0225 ± 0.0118^a^ | 23 |
| *Maesa insignis* | 0.0300 ± 0.0188 | 3 |
| *Manglietia fordiana* | 0.0165 ± 0.0160 | 15 |
| *Manglietia moto* | 0.0488 ± 0.0133 | 2 |
| *Melastoma affine* | 0.0429 ± 0.0308 | 5 |
| *Meliosma fordii* | 0.0206 ± 0.0083 | 3 |
| *Meliosma rigida Sieb. et Zucc. var. pannosa* | 0.0281 | 1 |
| *Meliosma squamulata* | 0.0181 ± 0.0137 | 15 |
| *Microtropis gracilipes* | 0.0219 ± 0.0213^a^ | 59 |
| *Myrica rubra* | 0.0174 | 1 |
| *Mytilaria laosensis* | 0.0178 ± 0.0132 | 2 |
| *Neolitsea aurata* | 0.1857 ± 0.1444 | 3 |
| *Neolitsea phanerophlebia* | 0.0232 ± 0.0387^a^ | 370 |
| *Olea dioica* | 0.0160 ± 0.0101 | 39 |
| *Ormosia fordiana* | 0.0148 ± 0.0118 | 4 |
| *Ormosia glaberrima* | 0.0245 ± 0.0262^a^ | 127 |
| *Ormosia pachycarpa* | 0.0231 ± 0.0153 | 8 |
| *Pentaphylax euryoides* | 0.0259 | 1 |
| *Photinia prunifolia* | 0.0187 ± 0.0182 | 9 |
| *Pithecellobium lucidum* | 0.0200 ± 0.0111 | 14 |
| *Pittosporum glabratum* | 0.0166 ± 0.0104 | 16 |
| *Pterospermum heterophyllum* | 0.0089 ± 0.0069 | 7 |
| *Randia canthioides* | 0.0214 ± 0.0170^a^ | 141 |
| *Rapanea neriifolia* | 0.0213 ± 0.0170^a^ | 281 |
| *Reevesia thyrsoidess* | 0.0222 ± 0.0215^a^ | 50 |
| *Rehderodendron macrocarpum* | 0.0240 ± 0.0144 | 11 |
| *Rhododendron simsii* | 0.0177 ± 0.0130 | 145 |
| *Saurauia tristyla* | 0.0099 | 1 |
| *Schefflera octophylla* | 0.0397 ± 0.0562^a^ | 13 |
| *Schima superba* | 0.0721 ± 0.1146 | 5 |
| *Schoepfia chinensis* | 0.0226 ± 0.0128 | 11 |
| *Sinosideroxylon wightianum* | 0.0196 ± 0.0162^a^ | 255 |
| *Sloanea sinensis* | 0.0343 ± 0.0450 | 8 |
| *Symplocos adenophylla* | 0.0453 ± 0.0842 | 6 |
| *Symplocos anomala* | 0.0244 ± 0.0242 | 15 |
| *symplocos congesta* | 0.0318 ± 0.0383 | 8 |
| *Symplocos lancifolia* | 0.0134 ± 0.0061 | 20 |
| *Symplocos laurina* | 0.0176 ± 0.0083 | 7 |
| *Symplocos stellaris* | 0.0126 ± 0.0052 | 2 |
| *Syzygium championii* | 0.0221 ± 0.0179^a^ | 27 |
| *Syzygium hancei* | 0.0225 ± 0.0149^a^ | 44 |
| *Syzygium kwangtungense* | 0.0207 ± 0.0138^a^ | 35 |
| *Tarenna mollissima* | 0.0137 ± 0.0079 | 13 |
| *Ternstroemia gymnanthera* | 0.0255 ± 0.0308^a^ | 99 |
| *Turpinia arguta* | 0.0232 ± 0.0217^a^ | 26 |
| *Tutcheria championi* | 0.0216 ± 0.0195^a^ | 49 |
| *Vitex quinata* | 0.0117 ± 0.0091 | 17 |
| *Wikstroemia nutans* | 0.0194 ± 0.0122 | 16 |
| *Xanthophyllum hainanense* | 0.0217 ± 0.0226^a^ | 312 |
| *Zanthoxylum myriacanthum* | 0.2697 ± 0.0130 | 2 |
