# Supplementary material for: Comparing shade tolerance measures of woody forest species
Source: PeerJ. 2018 Oct 9;6:e5736. doi: 10.7717/peerj.5736 (PMC6183557; doi:10.7717/peerj.5736)
Supplement: Supplemental Information 5 [file peerj-06-5736-s005.docx]

| **Species** | **Succession seral stages** | **Shade-tolerance/intolerance groups** |
| --- | --- | --- |
| *Altingia chinensis* | Later stage^a^ | - |
| *Antidesma venosum* | Later stage^a^ | - |
| *Ardisia elegans* | Middle stage^a^ | Shade tolerant |
| *Ardisia quinquegona* | Later stage^a^ | Shade tolerant^a^ |
| *Artocarpus styracifolius* | Later stage^a^ | Shade tolerant^a^ |
| *Camellia caudata* | Later stage^a^ | - |
| *Camellia sinensis* | Early stage^a^ | - |
| *Canarium album* | Later stage^a^ | - |
| *Carallia brachiata* | *-* | Shade intolerant |
| *Castanopsis carlesii* | Early stage^a^ | - |
| *Castanopsis fabri* | Middle stage^a^ | - |
| *Castanopsis fordii* | Early stage | Shade intolerant |
| *Castanopsis nigrescens* | Later stage^a^ | - |
| *Cinnamomum porrectum* | Early stage^a^ | Shade intolerant^a^ |
| *Cryptocarya concinna* | Later stage^a^ | Shade tolerant^a^ |
| *Cyclobalanopsis chungii* | Later stage^a^ | - |
| *Diospyros morrisiana* | Early stage^a^ | Shade intolerant^a^ |
| *Diplospora dubia* | Later stage^a^ | - |
| *Elaeocarpus chinensis* | Early stage^a^ | - |
| *Elaeocarpus decipiens* | Early stage^a^ | - |
| *Engelhardtia roxburghiana* | Later stage^a^ | - |
| *Evodia lepta* | Middle stage^a^ | Shade tolerant |
| *Garcinia multiflora* | Later stage^a^ | - |
| *Heteropanax brevipedicellatus* | Later stage^a^ | Shade tolerant |
| *Homalium cochinchinense* | Later stage^a^ | - |
| *Ilex memecylifolia* | Later stage^a^ | - |
| *Ilex pubilimba* | Middle stage^a^ | - |
| *Ilex rotunda* | Early stage^a^ | - |
| *Itea chinensis* | Later stage^a^ | Shade tolerant^a^ |
| *Ixonanthes chinensis* | Later stage^a^ | - |
| *Lindera chunii* | Later stage^a^ | Shade tolerant^a^ |
| *Lithocarpus litseifolius* | Early stage^a^ | Shade intolerant |
| *Lithocarpus lohangwu* | Later stage^a^ | Shade tolerant^a^ |
| *Lithocarpus uvariifolius* | Middle stage^a^ | - |
| *Litsea acutivena* | Later stage^a^ | - |
| *Litsea elongata* | Later stage^a^ | - |
| *Litsea rotundifolia var. oblongifolia* | Early stage^a^ | - |
| *Machilus breviflora* | Later stage^a^ | Shade tolerant^a^ |
| *Machilus velutina* | Later stage^a^ | Shade tolerant^a^ |
| *Manglietia fordiana* | *-* | Shade intolerant |
| *Manglietia moto* | Early stage^a^ | - |
| *Melastoma affine* | Early stage^a^ | - |
| *Meliosma fordii* | Middle stage^a^ | - |
| *Myrica rubra* | Early stage^a^ | - |
| *Neolitsea phanerophlebia* | Later stage^a^ | - |
| *Ormosia glaberrima* | Later stage^a^ | Shade tolerant^a^ |
| *Ormosia pachycarpa* | Later stage^a^ | - |
| *Pithecellobium lucidum* | Early stage^a^ | Shade intolerant^a^ |
| *Randia canthioides* | Later stage^a^ | - |
| *Rapanea neriifolia* | Later stage^a^ | - |
| *Schefflera octophylla* | Early stage^a^ | Shade intolerant^a^ |
| *Schima superba* | Early stage^a^ | Shade intolerant^a^ |
| *Sinosideroxylon wightianum* | Later stage^a^ | - |
| *Sloanea sinensis* | Early stage^a^ | - |
| *Symplocos adenophylla* | Middle stage^a^ | Shade intolerant^a^ |
| *Symplocos lancifolia* | Early stage^a^ | - |
| *Symplocos laurina* | Later stage^a^ | - |
| *Ternstroemia gymnanthera* | Later stage^a^ | - |
| *Turpinia arguta* | Early stage^a^ | - |
| *Tutcheria championi* | Early stage^a^ | - |
| *Xanthophyllum hainanense* | Later stage^a^ | - |

Items with superscript letters “a” refer to Zhou *et al* (1999), and ones without superscript letters refer to the *Flora of China.*
